# Steroid hormone binding and modulation of *Trichinella spiralis* progesterone receptor: A computational approach

**DOI:** 10.1016/j.bbrep.2025.102031

**Published:** 2025-05-05

**Authors:** Muhammad Tahir Aleem, Shakeel Ahmed Lakho, Muhammad Mohsin, Shahbaz Ul haq, Ashiq Ali, Danmei Huang, Fenfei Gao

**Affiliations:** a Department of Pharmacology, Shantou University Medical College, Shantou, 515041, China; b Center for Gene Regulation in Health and Disease, Department of Biological, Geological, and Environmental Sciences, College of Sciences and Health Professions, Cleveland State University, Cleveland, OH, 44115, USA; c Laboratory of Cancer Biology and Epigenetics, Department of Cell Biology and Genetics, Shantou University Medical College China, China; d Fujian University of Traditional Chinese Medicine, Fujian, Fuzhou, China; e Department of Histology and Embryology, Shantou University Medical College, 515041, Shantou, China

**Keywords:** Steroid hormones, *Trichinella spiralis*, *Ts-MAPRC2*, Molecular modeling, Simulation

## Abstract

Trichinellosis, caused by *Trichinella* species, including *Trichinella spiralis*, is a foodborne zoonotic disease. Upon ingestion, ML swiftly invade the intra-multicellular niche of the small intestine, undergoing four rapid molts to mature into adults. In general, the route of transmission for *T. spiralis* nematodes occurs through ingestion of pork. Therefore, it is crucial to develop a vaccine that can deal with trichinellosis, particularly for humans and pigs. In this study, homology modelling, molecular docking, simulations and molecular mechanics-based scoring (MM/GBSA) are used to investigate the interaction between *T. spiralis* membrane-associated progesterone receptor component 2 (*Ts*-MAPRC2) and different steroids hormones. In the most favorable region, 93 amino acid residues (92.1 %) are located, while 7 amino acids (6.9 %) are located in the allowed region, showing that the model with a 93.33 ERRAT quality factor is good quality. The MD simulations were conducted for 50 ns to explore the affinities and stability of four hormones chosen from the docking studies that showed similar binding poses to the control hormone Mifepristone. Simulations showed that the selected hormones were potent Ts-MAPRC2 binders and can act as leads to determine their activity by biophysical assays. Discovery of these five steroid hormones and interactions with Ts-MAPRC2 could lead to new therapies and vaccines for trichinellosis.

## Highlights

• Role of Steroid hormones in *Trichinella spiralis*.
• Steroid Hormone Binding and Modulation.
• *Trichinella spiralis* Progesterone Receptor.
• Structure Prediction and Validation.
• Molecular modeling; simulation.

## 1 Introduction

Trichinellosis in humans is caused by species of the *Trichinella* genus, including the most common species, *Trichinella spiralis (T. spiralis)* [1]. Generally, one of the most common sources of *T. spiralis* infection is undercooked or raw pork [[2], [3], [4]]. The rising prevalence of this disease in China correlates with the country's high consumption of pork and pork products [[5], [6], [7]]. *T. spiralis* survival primarily depends on pork ingestion, adapting to host immune responses and cellular functions throughout all infection stages [8,9]. It is common to buy antihelminthic agents for treating trichinellosis, but overuse can lead to residue in meat, parasite resistance, and other problems for the environment. An effective vaccine development against trichinellosis for both humans and pigs would be highly beneficial [7,10]. Recent research has discovered a number of proteins that act as vaccine candidates because they inhibit parasite viability and create immunity against parasite infestation. Moreover, their protection against *T. spiralis* larvae has been tested in animal models infected with larvae of this parasite [[11], [12], [13], [14]]. Although research efforts have explored vaccine development against *T. spiralis*, no commercially viable vaccine exists to date. However, given the current control measures, including meat inspection through artificial digestion, the practical implementation of a vaccine remains uncertain [1]. Membrane-associated progesterone receptors (MAPRs) are a family of membrane-bound proteins that consists of the progesterone receptor membrane component 1 (PGRMC-1) and two (PGRMC-2) [[15], [16], [17], [18]]. Also, androgen receptors are interacting with small proteins in several studies that can be found in parasite including S. *japonicum.* In helminths such as *S*. *japonicum* and *Taenia solium*, the presence of PGRMC receptors and progesterone-induced proteins has been documented [19,20].

Previous studies focused on the cloning and characterization of the Ts-MAPRC2 gene in *T*. *spiralis*. Throughout this process, comprehensive analyses were performed to evaluate the gene's properties [21]. Furthermore, we have investigated how progesterone (P4) and mifepristone (RU486) affect Ts-MAPRC2 gene expression in muscle larvae (ML), female adult worms (F-AL), and male adult worms (M-AL). On the basis of these findings, we assessed the relative mRNA and phenotypic effects of mifepristone on the F-AL stage in vivo.

The predicted and validated structure of the membrane-associated progesterone receptor component 2 (mPGRMC2) of *T. spiralis* exhibits 44.54 % sequence identity with human PGRMC1 (PDB ID: 4X8Y) [22]. A significant reduction in host larval survival and infection was additionally noted with the siRNA-Ts-MAPRC2 (siRNA180, siRNA419, siRNA559) [[21], [22], [23]]. The present study investigates the interactions of the Ts-MAPRC2 gene with various steroid hormones. Molecular docking and molecular dynamics simulations have been employed to assess the binding affinities between Ts-MAPRC2 and these hormones. Specifically, the potential antiparasitic effects of these steroid hormones against *T*. *spiralis* will be evaluated. To determine the binding free energies of the complex systems, molecular mechanics-based scoring methods, specifically MM/GBSA, have been utilized.

## 2 Methodology

### 2.1 Analyzing Ts-MAPRC2 homology

Ts-MAPRC2 gene was searched against the Protein Databank using the NCBI Blastp server (https://blast.ncbi.nlm.nih.gov/Blast.cgi) (NCBI accession no. XP_003375934.1) [24]. The three-dimensionality structure of the Ts-MAPRC2 protein was modeled by SWISS-MODEL (https://swissmodel.expasy.org/) using default parameters. Model quality was estimated using two parameters: GMQE and QMEAN. Additionally, SAVES Server data (https://saves.mbi.ucla.edu/) verified the quality of the model. Various algorithms can be employed to assess model validation, including PROCHECK [25], which evaluates the stereochemical quality of the model, and ERRAT, which analyzes the overall structural reliability [26]. In addition, VERIFY 3D (https://www.doe-mbi.ucla.edu/verify3d/) assessed the compatibility of protein structures by testing their association with non-covalently bonded amino acids.

### 2.2 Hormone retrieval and docking

A selection of steroids was made that would bind to MAPRC2. A predicted model of Ts-MAPRC2 was generated by the Protein Preparation Wizard in Maestro (Schrodinger's suite) [27]. In order to maintain structural integrity, hydrogen atoms were added to the receptor, amino acids were assigned bond orders, and metals were created with zero-order bonds. At 7.4 pH, tautomeric states and protonation were adjusted to refine the structure. A force field was applied to the structure to optimize its geometry, and then minimize its dimensions using OPLS_2005 [28]. A 3D grid at the cartesian coordinates using dimensions of 46.06, −13.52, and 60.33 for X, Y, Z was generated. Additionally, LigPrep was used to prepare all the ligands (hormones). At pH 7.0, epik was used to generate ionization states for ligands. OPLS_2005 force field was used to generate stereoisomers of ligands and low energy conformers.In molecular docking, conformers with low energies were used. In order to select the best poses, docked ligands were ranked according to their glide scores.

### 2.3 MD simulation

To check the stability of the selected hormones with the Ts-MAPRC2, a 50 ns long MD simulation was conducted by using the VMD [29] and NAMD [30]. AMBER21 tools were used to prepare the input files [31]. Adding hydrogen to proteins was accomplished using the LeaP program [32]. The TIP3P water model was used to solvate protein-ligand complexes in a periodic box of 10 Å [33]. To neutralize the systems prior to minimization, counterions of Na+ and Cl-were introduced. Proteins and ligands were analyzed using the ff14SB and gaff forcefields, respectively. A minimization at 10000 steps relaxed the systems to avoid energy clashes. Solvation system equilibrium was achieved at 310K after removing system clashes. The system was further equilibrated by increasing the temperature three times (from 200K to 250K and 300K) in order to maintain stability. While production was running, MD trajectories were stored after every 2 ps. We analyzed MD trajectories using VMD tcl commands, CPPTRAJ [34] and R package [35].

### 2.4 MM/GBSA analysis

MM/GBSA scoring methods were used to calculate binding free energies of the systems. Based on the last 2 ns stable MD trajectory, 300 snapshots of the complex were taken at 2 ps intervals. Binding free energy is calculated as the difference between total free energy and binding free energy (ΔG_com_) of the ligand-receptor complex as well as an individual receptor's free energy (ΔG_pro_) and ligand (ΔG_lig_) based on the equation provided below: ΔG_bind_ = ΔH - TΔS = ΔG_com_ - (ΔG_pro_ + ΔG_lig_) [22].

## 3 Results

### 3.1 Structure Prediction and Validation

**Fig. 1:**
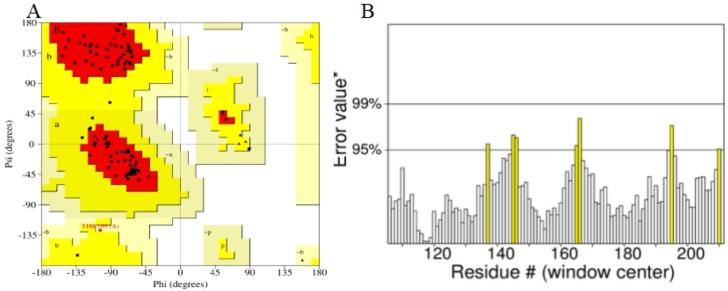
(A) Ramachandran Plot: The plot reveals that 92.1 % of the residues are located within the most favored regions (depicted in red), while 6.9 % of the residues fall within additionally allowed regions (yellow). Notably, no residues were found in the disallowed regions (white), indicating favorable stereochemical quality of the modeled structure. (B) ERRAT Quality Factor: The ERRAT quality chart was used to assess the overall model reliability based on non-bonded atomic interactions.

The membrane-associated progesterone receptor component 2 (MAPRC2) of *Trichinella spiralis* exhibited a sequence identity of 44.54 % with the human progesterone receptor membrane component 1 (PGRMC1; PDB ID: 4X8Y). Homology modeling of Ts-MAPRC2 yielded a Global Model Quality Estimation (GMQE) score of 0.36 and a Qualitative Model Energy Analysis (QMEAN) score of 0.64. The GMQE score, which integrates information from the target-template alignment and template selection process, ranges from 0 to 1, with higher values indicating increased reliability of the predicted model. A QMEAN score near zero suggests that the predicted model is in good agreement with high-resolution experimental structures of similar size, whereas scores below −4.0 typically indicate poor model quality. Structural validation of the predicted three-dimensional (3D) model was performed using PROCHECK by generating a Ramachandran plot (Fig. 1A). The analysis showed that 93 residues (92.1 %) were located within the most favored regions, while 7 residues (6.9 %) fell within the additionally allowed regions. Notably, no residues were found in disallowed regions, indicating favorable stereochemical quality of the model. Furthermore, an ERRAT analysis yielded an overall quality factor of 93.33 (Fig. 1B), supporting the high reliability of the constructed homology model.

### 3.2 Molecular docking

**Fig. 2:**
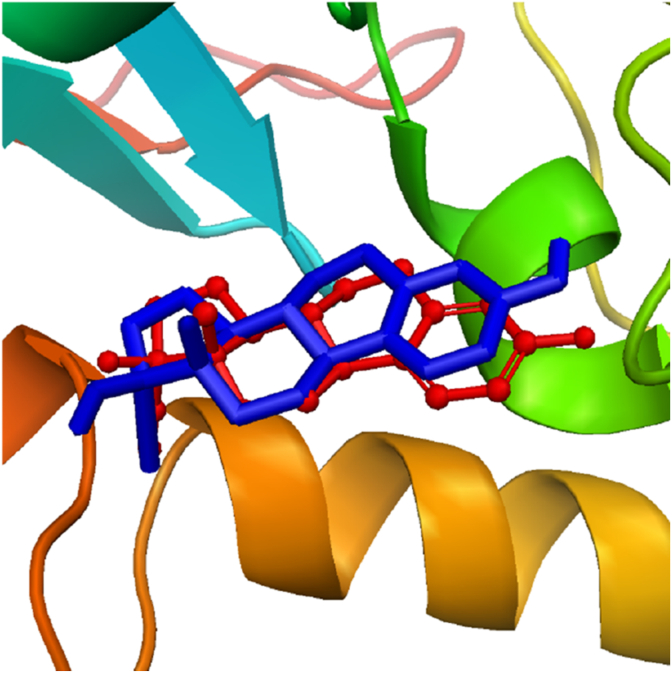
The re-docking of 17β-estradiol into the protein binding pocket. The blue stick representation denotes the initially docked conformation, while the red ball-and-stick model represents the re-docked conformation.

The prepared hormones were docked onto the generated grid of Ts-MAPRC2 to determine their binding poses. The reliability of the docking protocol was validated through the redocking of 17β-estradiol, which demonstrated that the ligand was docked in the same binding pocket location (Fig. 2). The docking analysis revealed varying binding affinities among the hormones, with glide scores ranging from −3.01 to −6.65 kcal/mol. The binding affinities and corresponding 2D structures of the hormones are presented in Table 1.

**Table 1:** The structures and binding affinities of the hormones with Ts-MAPRC2.

No.	Hormones	Structures	Glide score
1	17-estradiol		−6.655
2	Norepinephrine		−6.052
3	Prostaglandins		−4.940
4	Aglepristone		−4.943
5	Mifepristone		−4.391
6	Ulipristal		−4.067
7	Progesterone		−3.732
8	Cortisol		−3.710
9	Dehydroepiandrosterone		−3.349
10	Testosterone		−3.086
11	Corticosterone		−3.034
12	Dexamethasone		−3.019

### 3.3 Analyzing molecular docking

**Fig. 3:**
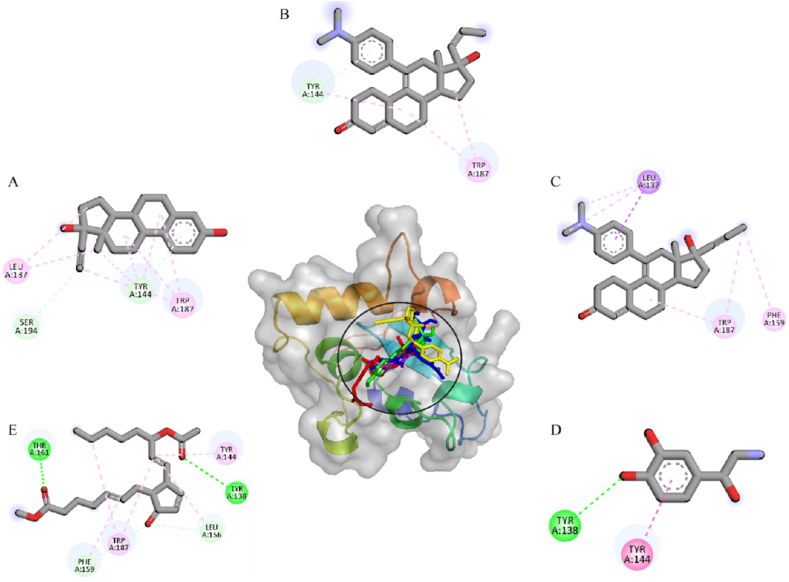
The binding modes and interactions of the selected hormones. (A) 17-Estradiol (B) Aglepristone (C) Mifepristone (D) Norepinephrine (E) Prostaglandins. Subsequently, these hormones were docked into the predicted binding site of the target protein Ts-MAPRC2 to assess potential interactions.The three-dimensional structure is displayed at the center, offering a comprehensive representation of the interactions involved.

A total of five hormones were selected for analysis based on their binding modes and affinities. These hormones were subsequently docked into the predicted binding pocket of the target protein (Ts-MAPRC2). The selected hormones included 17β-estradiol, aglepristone, mifepristone, norepinephrine, and prostaglandins. Molecular docking analysis revealed that 17β-estradiol formed two hydrogen bonds with Tyr144 and Ser194, along with two alkyl interactions involving Leu137 and Trp187 (Fig. 3A). Aglepristone established one hydrogen bond with Tyr144 and a single alkyl interaction with Trp187 (Fig. 3B). Mifepristone exhibited one sigma interaction with Leu137 and two alkyl interactions with Phe159 and Trp187 (Fig. 3C). Similarly, norepinephrine formed one hydrogen bond with Tyr138 and one alkyl interaction with Tyr144 (Fig. 3D). Lastly, prostaglandins established three hydrogen bonds with Thr161, Tyr138, and Leu156, in addition to two alkyl interactions with Tyr144 and Trp187 (Fig. 3E). The binding modes within the protein's binding pocket are depicted in Fig. 3, along with the associated molecular interactions. The 3D structure is presented at the center, providing a detailed representation of these interactions.

### 3.4 MD simulation outcomes

A molecular dynamics (MD) simulation was conducted to evaluate the stability and binding affinity of hormones with Ts-MAPRC2. To assess the MD trajectories of the selected complexes, various structural parameters, including root-mean-square deviation (RMSD), root-mean-square fluctuation (RMSF), radius of gyration (Rg), and solvent-accessible surface area (SASA), were analyzed through respective plots.

#### 3.4.1 RMSD result

**Fig. 4:**
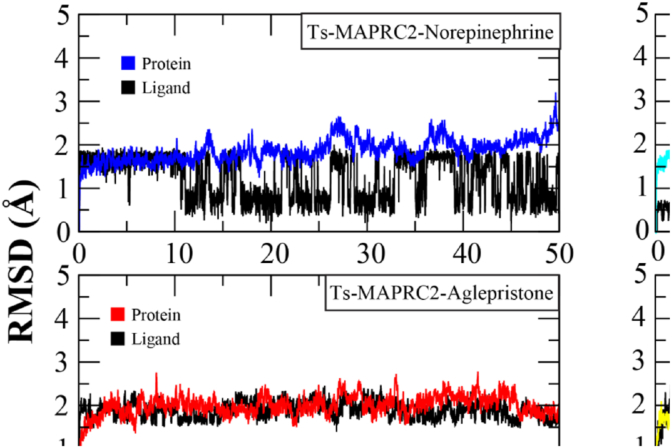
The RMSD plots of backbone of Ts-MAPRC2 protein complexed with hormones. The RMSD plots of ligands docked with the Ts-MAPRC2protein. All systems are distinguished by color coding as follows: blue for Ts-MAPRC2–Norepinephrine, red for Aglepristone, turquoise for 17β-Estradiol, yellow for Prostaglandins, green for Mifepristone, and black for the ligand.

In a 50 ns molecular dynamics simulation, the root-mean-square deviation (RMSD) of the ligand-bound protein backbone was analyzed to evaluate the structural stability of five protein-ligand complexes. The RMSD trajectories of these complexes are depicted in Fig. 4. All complexes reached equilibrium within the initial 5 ns and maintained a stable RMSD range of approximately 2.0–2.5 Å up to 25 ns. However, deviations in RMSD values were observed beyond this time point. Among the analyzed complexes, the Ts-MAPRC2-Mifepristone complex (green) exhibited the most pronounced fluctuations, with its RMSD increasing to ∼4.0 Å at 30 ns. This complex displayed significant deviations between 30 and 40 ns before stabilizing at approximately 4.0 Å after 40 ns. The mean RMSD for this complex was calculated as 2.64 ± 0.8 Å. In contrast, the Ts-MAPRC2-Prostaglandins complex experienced only minor fluctuations between 25 and 30 ns, momentarily reaching ∼3.5 Å before returning to its initial conformational stability (2.0–2.5 Å) after 30 ns, with an average RMSD of 2.32 ± 0.3 Å. Of the remaining complexes, only Ts-MAPRC2-17-Estradiol exhibited deviations towards the later stages of the simulation. This complex maintained structural stability until 45 ns, after which its RMSD increased to ∼4.0 Å. The average RMSD for this complex was determined to be 1.98 ± 0.3 Å. Conversely, the Ts-MAPRC2-Norepinephrine and Ts-MAPRC2-Aglepristone complexes remained stable throughout the entire simulation, with mean RMSD values of 1.98 ± 0.2 Å and 1.97 ± 0.2 Å, respectively. To further assess the stability of protein-ligand interactions, the RMSD of the docked ligands was also examined (Fig. 4). The analysis indicated that all ligands remained within the ideal RMSD range of approximately 2.0 Å, signifying stable binding throughout the simulation. Among these, the Ts-MAPRC2-17-Estradiol complex exhibited the highest stability, with an average ligand RMSD of 0.54 ± 0.1 Å. Conversely, the Ts-MAPRC2-Prostaglandins complex displayed the highest ligand RMSD, with an average value of 1.91 ± 0.2 Å.

#### 3.4.2 RMSF analysis

**Fig. 5:**
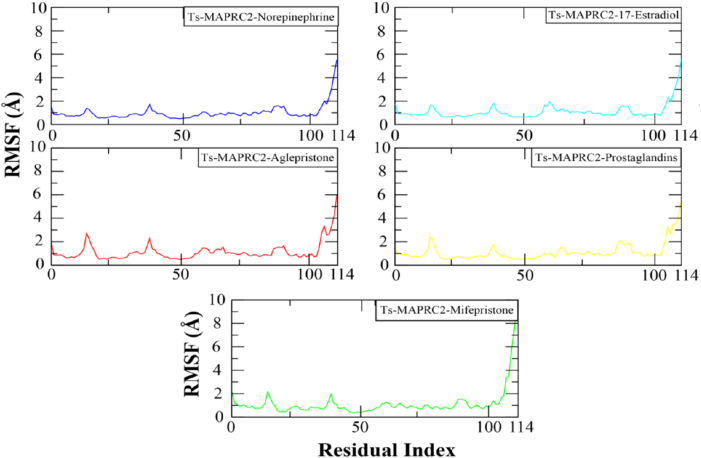
Simulating Ts-MAPRC2 complexes and analyzing residual flexibility.Each system is distinguished by a specific color code: blue for Ts-MAPRC2-Norepinephrine, red for Ts-MAPRC2-Aglepristone, turquoise for Ts-MAPRC2-17β-Estradiol, yellow for Ts-MAPRC2-Prostaglandins, and green for Ts-MAPRC2-Mifepristone.

The Root Mean Square Fluctuation (RMSF) analysis was conducted to evaluate the dynamic behavior of amino acid residues in the protein upon hormone binding during molecular dynamics simulations. Regions exhibiting high RMSF values correspond to flexible structural elements, such as loops, whereas lower RMSF values are indicative of more rigid secondary structures, including α-helices and β-sheets. The RMSF profiles of the five simulated systems are depicted in Fig. 5. Notably, substantial fluctuations were predominantly observed in loop regions. In particular, residues 9–19, which constitute a loop, displayed an RMSF value of approximately 2.5 Å. In contrast, residues exhibiting lower RMSF values were likely associated with α-helical and β-sheet structures. Additionally, the highest RMSF values were recorded for residues 104–114, which correspond to an extended loop at the C-terminal region.

#### 3.4.3 Resulting radius of gyration

**Fig. 6:**
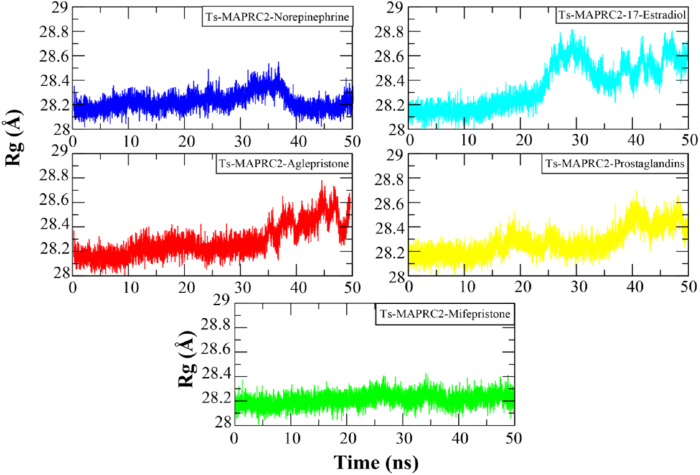
The protein compactness analysis of Ts-MAPRC2 complexes by calculating Radius of Gyration. Each system is distinguished by a specific color code: blue for Ts-MAPRC2-Norepinephrine, red for Ts-MAPRC2-Aglepristone, turquoise for Ts-MAPRC2-17β-Estradiol, yellow for Ts-MAPRC2-Prostaglandins, and green for Ts-MAPRC2-Mifepristone.

The compactness of the protein in all complexes was assessed using Radius of Gyration (Rg) analysis. An increase in Rg values indicates protein unfolding during the simulation. Fig. 6 presents the Rg plots of Ts-MAPRC2 complexes, which exhibited a trend similar to the RMSD plots. Specifically, the Rg values remained stable during the initial 25 ns of the simulation. Across all trajectories, the Rg values ranged between approximately 28.1 and 28.4 Å. After 25 ns, the Ts-MAPRC2-17-Estradiol complex exhibited a slight deviation, reaching a maximum Rg value of ∼28.8 Å, which persisted until the end of the simulation. Other complexes also displayed minor fluctuations within a range of 0.2 Å. Overall, the systems remained stable throughout the simulation, and no significant protein unfolding events were observed.

#### 3.4.4 Analysis of solvent accessible surface area (SASA)

**Fig. 7:**
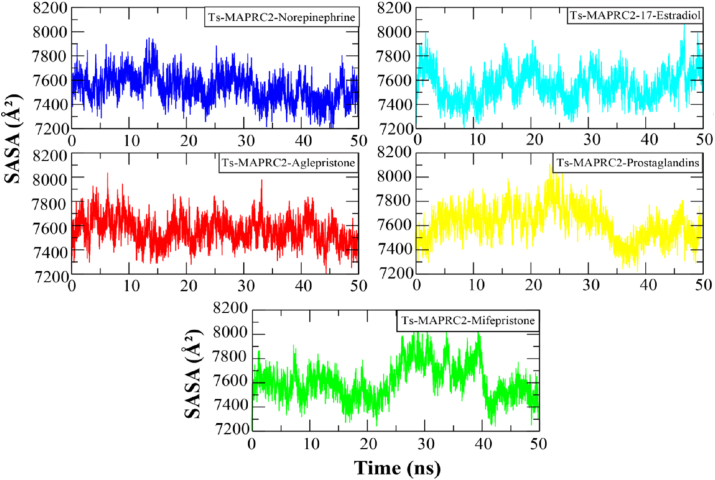
The solvent-accessible surface area calculation of Ts-MAPRC2 complexes.Each system is distinguished by a specific color code: blue for Ts-MAPRC2-Norepinephrine, red for Ts-MAPRC2-Aglepristone, turquoise for Ts-MAPRC2-17β-Estradiol, yellow for Ts-MAPRC2-Prostaglandins, and green for Ts-MAPRC2-Mifepristone.

The solvent-accessible surface area (SASA) of the Ts-MAPRC2 complexes was calculated to assess the protein surface exposed to the solvent in the presence of bound hormones. The results revealed that the compact system exhibited a lower solvent-exposed surface area, whereas higher SASA values indicated distortions in the protein structure. Fig. 7 presents the SASA plots for the complexes, expressed in Å^2^. Notably, the Ts-MAPRC2-Prosaglandin complex displayed significant irregularities in its SASA profile, with a peak value at 25 ns, followed by a continuous decrease from approximately 8100 Å^2^ to 7000 Å^2^ at 40 ns. By the end of the simulation, the SASA value stabilized at around 7600 Å^2^. In contrast, the other complexes did not exhibit such notable fluctuations in their SASA values. Overall, the SASA data indicate that the complexes remained structurally compact, without significant distortions occurring in the protein structure.

### 3.5 Calculation of the binding free energy

The binding free energies of the Ts-MAPRC2 complex were computed using the MM/GBSA module of Amber 21. The binding free energy was calculated from the last 300 snapshots of the molecular dynamics (MD) trajectory. To obtain the binding free energies, the total energies of the receptor and ligand were subtracted from the total energy of the complex. As presented in Table 2, all complexes exhibit identical binding free energies.

**Table 2:** The binding free energy calculations of Ts-MAPRC2 complexes.

	17-Estradiol	Norepinephrine	Aglepristone	Mifepristone	Prostaglandins
ΔE_vdW_	−33.29 ± 0.27	−22.31 ± 0.22	−37.62 ± 0.32	−54.01 ± 0.30	−47.50 ± 0.34
ΔE_ele_	−1.11 ± 0.75	−3.29 ± 0.17	51.55 ± 1.11	0.00 ± 0.00	0.00 ± 0.00
ΔE_GB_	4.22 ± 0.76	9.50 ± 0.17	−41.68 ± 1.11	8.33 ± 0.08	6.55 ± 0.07
ΔE_surf_	−3.95 ± 0.01	−3.03 ± 0.02	−4.23 ± 0.02	−4.42 ± 0.16	−5.79 ± 0.03
ΔG_gas_	−34.40 ± 0.82	−25.60 ± 0.29	13.92 ± 1.26	−54.01 ± 0.30	−47.50 ± 0.34
ΔG_solv_	0.26 ± 0.75	6.47 ± 0.16	−45.91 ± 1.10	3.90 ± 0.09	0.75 ± 0.05
TΔS	−15.96 ± 1.39	−13.47 ± 1.16	−21.06 ± 0.76	−19.37 ± 1.16	−26.74 ± 0.73
ΔG_total_	−34.13 ± 0.29	−19.13 ± 0.20	−31.98 ± 0.30	−50.10 ± 0.33	−46.74 ± 0.34

TΔS = entropy.

### 3.6 Energy decomposition

**Fig. 8:**
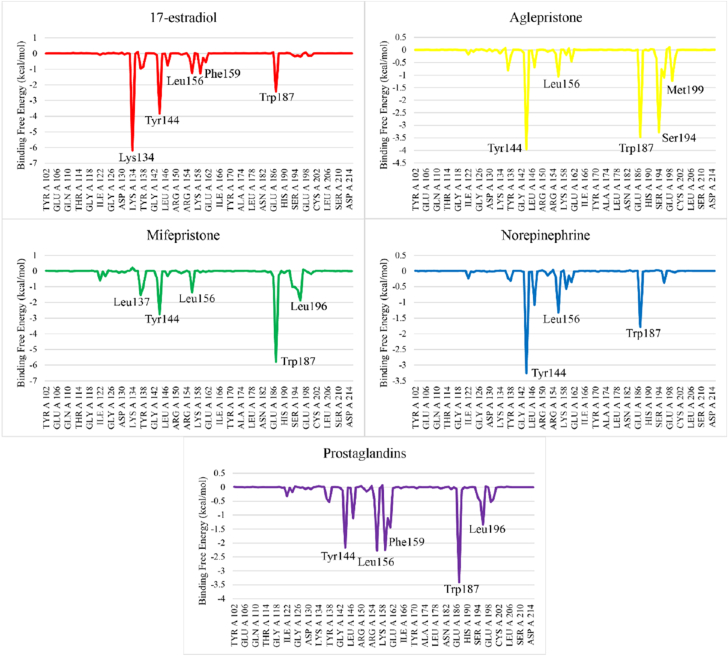
Binding energy decomposition analysis of the complexes to identify key interacting residues.

The residual contribution to the total binding energy was determined through energy decomposition analysis. Key residues involved in the binding interactions of the protein with 17-estradiol included Lys134, Tyr144, Leu156, Phe159, and Trp187. In the Aglepristone complex, the amino acids Tyr144, Leu156, Trp187, Ser194, and Met199 exhibited significant contributions to the binding free energy. For the Mifepristone complex, the residues Leu137, Tyr144, Leu156, Trp187, and Leu196 were found to make substantial contributions. Similarly, in the Norepinephrine complex, Tyr144, Leu156, and Trp187 contributed significantly to the binding energy, while in the Prostaglandin complex, the primary interacting residues included Tyr144, Leu156, Phe159, Trp187, and Leu196 (Fig. 8).

## 4 Discussion

Progesterone receptor membrane component-1 (PGRMC-1) and PGRMC-2 are key constituents of the membrane-associated progesterone receptor (MAPR) family. In helminths, including *S. japonicum*, progesterone-responsive proteins and PGRMC receptors have been characterized [[15], [16], [17], [18], [19], [20]]. A potentially functional progesterone receptor membrane Component (PGRMC) has been identified in *T. solium* and is characterized in this study. However, its evolutionary origin, whether derived through horizontal gene transfer, molecular mimicry, or inheritance from a common ancestor, remains unresolved [20]. In this study, we investigate the binding affinities and stabilities of various steroid hormones in relation to their interaction with the Ts-MAPRC2 protein using molecular modeling and simulation techniques (Table 1). The homology model of Ts-MAPRC2 was generated using Swiss Model, and its accuracy was evaluated through Ramachandran plot analysis and Errat quality scores (Fig. 1). The Ramachandran plot revealed that most of the amino acid residues in the model fall within the allowed regions, indicating that the model is of high quality. This suggests that the constructed model is reliable for further structural and functional analysis [22,26,36]. Further, the docking of the prepared hormones onto the Ts-MAPRC2 grid was performed to evaluate their binding poses. The protocol's accuracy was confirmed by redocking 17β-estradiol, which reproduced the same binding pocket, validating the approach, and subsequent docking of various steroid hormones retrieved from literature further supported the model's reliability using the Glide tool (Fig. 2). These findings suggest that Ts-MAPRC2 may serve as a promising target for hormone-based therapeutic interventions in parasitic infections [37]. To further explore potential interactions, we docked the hormones into the predicted binding site of the target protein Ts-MAPRC2. The resulting 3D structure, shown at the center, provides a detailed visualization of the interactions, allowing for a deeper understanding of the molecular dynamics at play. Moreover, the binding affinities of the selected hormones for the protein varied between −6.655 and −3.019 kcal/mol, indicating a range of binding strengths. Based on these docking scores and the observed binding orientations, five hormones 17-estradiol, aglepristone, mifepristone, norepinephrine, and prostaglandins were chosen for subsequent stability analysis. Mifepristone, known for its inhibitory activity against Ts-MAPRC2, was included as a control to assess the relative potency of the other hormones [21]. Thereafter, the binding sites of the selected hormones were consistently located within regions containing the following residues: Leu137, Tyr138, Tyr144, Phe147, Leu156, Phe159, Ser160, Thr161, Trp187, Ser194, and Tyr200. These residues form a network of interactions that stabilize the binding of the hormones to the protein. Specifically, the interactions included hydrogen bonds, pi-pi stacking, pi-alkyl stacking, and hydrophobic interactions, which collectively contribute to the binding affinity and specificity of each hormone (Fig. 3). The diversity of these interaction types suggests a robust and stable interaction profile, with the potential for significant functional implications in the inhibition of Ts-MAPRC2 activity [22]. To further validate our docking results, we conducted molecular dynamics simulations to investigate the stability and dynamic behavior of the hormone-Ts-MAPRC2 complexes over time. The 50 ns simulation revealed that the majority of the complexes maintained stable root mean square deviation (RMSD) values, indicating consistent binding behavior. However, the Ts-MAPRC2-Mifepristone complex exhibited significant deviations after 25 ns, suggesting potential instability or alterations in the binding pose of Mifepristone. Despite these deviations, the root mean square fluctuation (RMSF) analysis revealed minimal structural fluctuations, indicating that the protein itself remained largely stable throughout the simulation. Further, calculations of the radius of gyration and solvent-accessible surface area (SASA) confirmed that the protein-ligand complexes remained compact and structurally intact, highlighting the robustness of the complexes (Fig. 4, Fig. 5, Fig. 6, Fig. 7). These results reinforce the idea that the stability and compactness of the complexes, particularly in the presence of specific ligands like Mifepristone, are essential for preserving their functional integrity. This insight aligns with our docking predictions and provides a more comprehensive understanding of the molecular interactions governing these complexes [21,22,38]. Consequently, MM/GBSA calculations revealed binding free energies of −26.57 ± 0.16, −14.10 ± 0.13, −35.06 ± 0.23, −27.65 ± 0.31, and −32.12 ± 0.55 kcal/mol for Ts-MAPRC2 complexes with Estradiol, Norepinephrine, Aglepristone, Prostaglandins, and Mifepristone, respectively. These results indicate varying binding affinities, suggesting potential for selective targeting (Table 2) [22,38]. All four hormones demonstrated superior stability compared to the reference Ts-MAPRC2-Mifepristone complex, with their binding energies being comparable to Mifepristone. These findings suggest their potential as lead compounds for targeting Ts-MAPRC2 in biophysical assays, as further confirmed by MM/GBSA analysis [22,39]. Meanwhile, energy decomposition analysis revealed that key residues involved in the binding interactions varied across different complexes (Fig. 8). In particular, residues such as Tyr144, Leu156, and Trp187 consistently contributed significantly to the binding energy, with specific additional contributions from Lys134, Phe159, Ser194, and Met199 depending on the ligand. These findings highlight the crucial role of these residues in mediating the stability and specificity of ligand binding, suggesting potential targets for modulating protein-ligand interactions [22,24,28,39].

## 5 Conclusions

Different steroid hormones were retrieved from literature and docked against the *Ts*-MAPRC2 to get their binding modes. Based on the docking studies, four hormones were selected that showed similar binding poses like the control hormone Mifepristone and their binding affinities and stability with the *Ts*-MAPRC2 was explored by 50 ns long MD simulations. The results of simulations confirmed the selected hormones are potent binders of *Ts*-MAPRC2 and can act as lead to find their activity by biophycial assays. It is anticipated that the discovery of these five steroid hormones, as well as how they interact with Ts-MAPRC2, may lead to new therapies and vaccines for trichinellosis. A further study is under way to determine the in vitro and in vivo hormonal effects of this protein (Ts-MAPRC2).

## CRediT authorship contribution statement

**Muhammad Tahir Aleem:** Writing – review & editing, Writing – original draft, Visualization, Validation, Supervision, Resources, Investigation, Formal analysis, Data curation, Conceptualization. **Shakeel Ahmed Lakho:** Writing – review & editing, Visualization, Validation, Investigation, Formal analysis, Data curation. **Muhammad Mohsin:** Writing – review & editing, Visualization, Validation, Investigation, Data curation. **Shahbaz Ul haq:** Writing – review & editing, Visualization, Validation, Investigation, Data curation. **Ashiq Ali:** Writing – review & editing, Visualization, Validation, Investigation, Formal analysis. **Danmei Huang:** Writing – review & editing, Visualization, Validation, Resources, Investigation, Formal analysis. **Fenfei Gao:** Writing – review & editing, Visualization, Validation, Supervision, Investigation, Formal analysis, Data curation.

## Informed consent statement

Not applicable.

## Institutional review board statement

Not applicable.

## Disclosure statement

No potential conflict of interest was reported by the author(s).

IRB approval.

As this study does not use samples of human origin, it is not subject to IRB approval or a declaration of compliance with the Declaration of Helsinki.

## Funding

Shantou University Medical College project no. (350 510859066), China.

## Declaration of competing interest

The authors declare that there is no conflict of interest regarding the publication of this article.

## Data Availability

No data was used for the research described in the article.
